# Frequency of resuscitation attempts with dying nursing home residents. A full survey in an urban district in Germany based on registry data from 2018–2021

**DOI:** 10.1016/j.resplu.2023.100508

**Published:** 2023-11-13

**Authors:** Andreas Günther, Sybille Schmid, Uta Weidlich-Wichmann, Eileen Czaputa, Martina Hasseler, Jan Weber

**Affiliations:** a Institute for General Practice and Palliative Care, Hannover Medical School, Hanover, Germany; b Fire Department, City of Braunschweig, Eisenbütteler Straße 2, 38122 Braunschweig, Germany; c Faculty of Health and Health Care Sciences, Ostfalia University of Applied Sciences, Wolfsburg, Germany; d Social Services Department, City of Braunschweig, Braunschweig, Germany

**Keywords:** Nursing home, Cardiopulmonary resuscitation, Place of death, Sex difference, Overuse, Underuse

## Abstract

**Aim:**

The realities of emergency care and resuscitation research involving nursing home (NH) residents suggest an overuse of resuscitation attempts in NHs. A complete analysis of all NH resident deaths is needed to provide a complementary perspective of potential underuse. The present research investigated whether residents of different NH homes died at the NH during attempted resuscitation or after transfer to hospital.

**Methods:**

A full survey of resuscitation attempts and deaths among NH residents, via retrospective analysis of data from the death registry and the German Resuscitation Registry for the years 2018 to 2021.

**Results:**

Over the 4-year study period, 14,598 individuals died, of whom 3,288 (22.5%) were residents of 31 different NHs. The mean age of the deceased NH residents was 87 years (±8.6); 2,196 (66.8%) were female, 118 (3.6%) underwent a resuscitation attempt, and 58.5% died at the NH. NH averages were as follows: deaths per NH: 106 (±51; min–max: 36–292); number of beds: 102 (±39; 34–210); deaths per bed per year 0.27 (±0.07; 0.15–0.51); resuscitation attempts per 1,000 beds per year: 9.5 (±5.5; 0–21.1); and ratio of futile resuscitation attempts to deaths: 6.0% (0–12.5%). Considering the entire study region before and during the COVID-19 pandemic, a slight underuse of resuscitation attempts with female NH residents emerged. On a facility level, substantial disparities and opposing trends were found. The incidence of deaths and resuscitation attempts, as well as the place of death and the ratio of futile resuscitation attempts to deaths, varied considerably.

**Conclusion:**

Resuscitation attempts are rarely administered to dying NH residents. However, their frequency varies considerably between NHs.

## Introduction

Cardiopulmonary resuscitation is rarely attempted in nursing homes (NH) and, when it is attempted, it rarely does any good.^1^ While many people seem aware of the poor prognosis for attempted cardiopulmonary resuscitation (aCPR) in NHs, fewer seem aware of its relative infrequency in this setting. Furthermore, since the responsibility for declaring death usually falls outside the purview of emergency medical services (EMS), contact between EMS personnel and deceased NH residents is uncommon. Data show that, in NHs, EMS personnel mainly encounter resident fatalities in the context of aCPR.[2], [3] Thus, EMS personnel might perceive resuscitation attempts to be relatively common during NH residents’ final moments of life.

In support of this impression, most of the literature on cardiac arrests in NHs refers to patients who received aCPR by an out-of-hospital medical team.^2^ In comparison to community-dwelling patients, NH residents have less favorable prognoses for cardiac arrest.[4], [5] Studies involving NH residents from Denmark^6^, France^2^, Germany^5^, Korea^7^ and Victoria, Australia^8^ have reported survival to hospital discharge rates following aCPR at approximately 2%.

Despite possible survival rates in excess of 1%, aCPR for NH residents may be perceived as inappropriate and overused. However, the evidence does not necessarily support this assessment. Equally important to consider is the potential for underuse. Indeed, both overuse and underuse—referring to the delivery of unnecessary services and the failure to deliver needed services, respectively—are global concerns that can simultaneously affect the same healthcare system, organisation or patient.[9], [10] Evidence on the underuse of aCPR in NH residents is currently limited, as is information about the general frequency with which aCPR is administered to NH residents at end of life. In a cross-sectional study of NHs based in Belgium, England, Finland, Italy, the Netherlands and Poland aCPR was observed quite rarely. Its highest frequency was in England, where 5.5% of dying NH residents were administered the procedure.^11^ Additionally, in one German city, 4% of the NH residents who died at their home, were observed to have received aCPR prior to death.^12^.

Any enquiry into the prevalence of prehospital aCPR among NH residents at the end of life should consider only residents who died within the NH facility. This is because the transfer of dying NH residents to a hospital shifts the decision-making about aCPR from the NH to the hospital. While some research on the transfer of dying residents from the NH to the hospital and their place of death (PoD) has been published, many open questions remain.[13], [14], [15]

In this context, the significance of the PoD lies in its influence on the frequency of prehospital aCPR.

Since the deaths of NH residents unrelated to aCPR are neither a primary focus of research nor a common experience for EMS personnel, the present study aimed at contributing to a broader view, expanding the perspective from death during aCPR to consider all deaths of NH residents within various NH settings. Thus, the research considered whether residents of different NH died after being transferred to hospital or in their NH facility and whether prehospital aCPR was attempted. Additionally, it explored potential changes in these patterns during the Covid-19 pandemic.

## Methods

The present research was based on a retrospective analysis of data from the death registry and the German Resuscitation Registry, covering the years 2018 to 2021. The study region was the city of Braunschweig, located in Lower Saxony in Germany. Braunschweig encompasses 192 km^2^ and has a population of approximately 250,000. The average age is 43.5 years and 21% of residents are aged 65 years or older. The city is home to 31 residential long-term care facilities, which collectively accommodate approximately 3,100 individuals.^12^ These NH are characterized by continuous presence of nursing staff and sporadic visits by physicians.

### Death registry data

Residents of various NHs were identified in the death registry data through a comparison of the residential addresses of the deceased with the addresses of the various NHs. To ensure an exact identification, missing address details were investigated in the population register. PoD was categorized into five groups, with reference to the death certificate of Lower Saxony. The categories “at home” and “in nursing home” were merged to “NH,” while the categories “transport” and “other places” were combined into “other.” The category “hospital” was retained. Thus, this research classified all PoDs as “NH,” “hospital” or “other.”^16^.

### Resuscitation attempts

The EMS of the city of Braunschweig has been prospectively collecting data about aCPR since the year 2011. This data collection process is linked to the German Resuscitation Registry and overseen by the Medical Director of the EMS. Data for each aCPR event includes the location, (e.g. NH).[3], [17] Thus, each aCPR event at a care facility was assigned to the respective NH, via the NH address.

### Nursing homes

Residential long-term care facilities and their bed capacity were identified using data from the local authorities. To ensure the anonymity of each of the investigated NHs, no specific facility data was reported here. The size of each NH was classified according to bed capacity, using the following categories: “up to 90,” “91 to 120,” and”over 120” beds. NH ownership was classified as “public,” “non-profit,” “private-local” or “private-nationwide,” based on information from the facility’s online presence.[12], [18], [19].

### Indicators

In addition to the number of beds, deaths, and aCPR events linked with each NH, various indicators were generated for the NH resident population across the study region and within individual NH facilities. These included: (a) deaths per bed per year, (b) PoD distribution as a percentage of all deaths, and (c) aCPR incidence per 1,000 beds per year. The analysis also calculated the correlation between aCPR and death, as a measure of overuse.[17], [20] An absolute risk (AR) indicator was defined as the ratio of resuscitation attempts without survival to hospital discharge to deaths at the PoD “NH.”.

### Data processing, pandemic period and reference period

The data processing, anonymization and analysis followed established standards for secondary data analysis.[12], [21] A descriptive analysis of all deaths and aCPR events was conducted both by year and separately for the COVID-19 pandemic period (i.e., March 1, 2020, the month in which the first COVID-19 case was reported in the study region, to December 31, 2021) and a corresponding reference period (i.e., March 1, 2018 to December 31, 2019). A risk difference (RD) for aCPR was calculated to assess changes in the risk of receiving aCPR at end of life over the study period.

The study comprised part of a larger interdisciplinary research project (acronym”NOVELLE”) that aimed at developing and implementing emergency management guidelines for NHs. Ethical approval was granted by Ostfalia University of Applied Sciences on July 2, 2020. Data processing and analysis were performed using the software packages MS Access, MS Excel, and IBM SPSS Statistics 28.

## Results

In the study region 14,598 individuals died between 2018 and 2021, including 3,288 (22.5%) residents of long-term care facilities. Of these NH residents, 1,925 (58.5%) NH-residents died at PoD „NH“ and 1347 (41.0%) died at the PoD „hospital“. The mean age of the deceased NH residents was 87 years (*SD* ± 8.6), and 2,196 (66.8%) were female. Seventy (2.1%) individuals were younger than 65 years, 1,082 (32.9%) were aged between 65 and 85 years, and 2,136 (65.0%) were at least 85 years old.

**Fig. 1:**
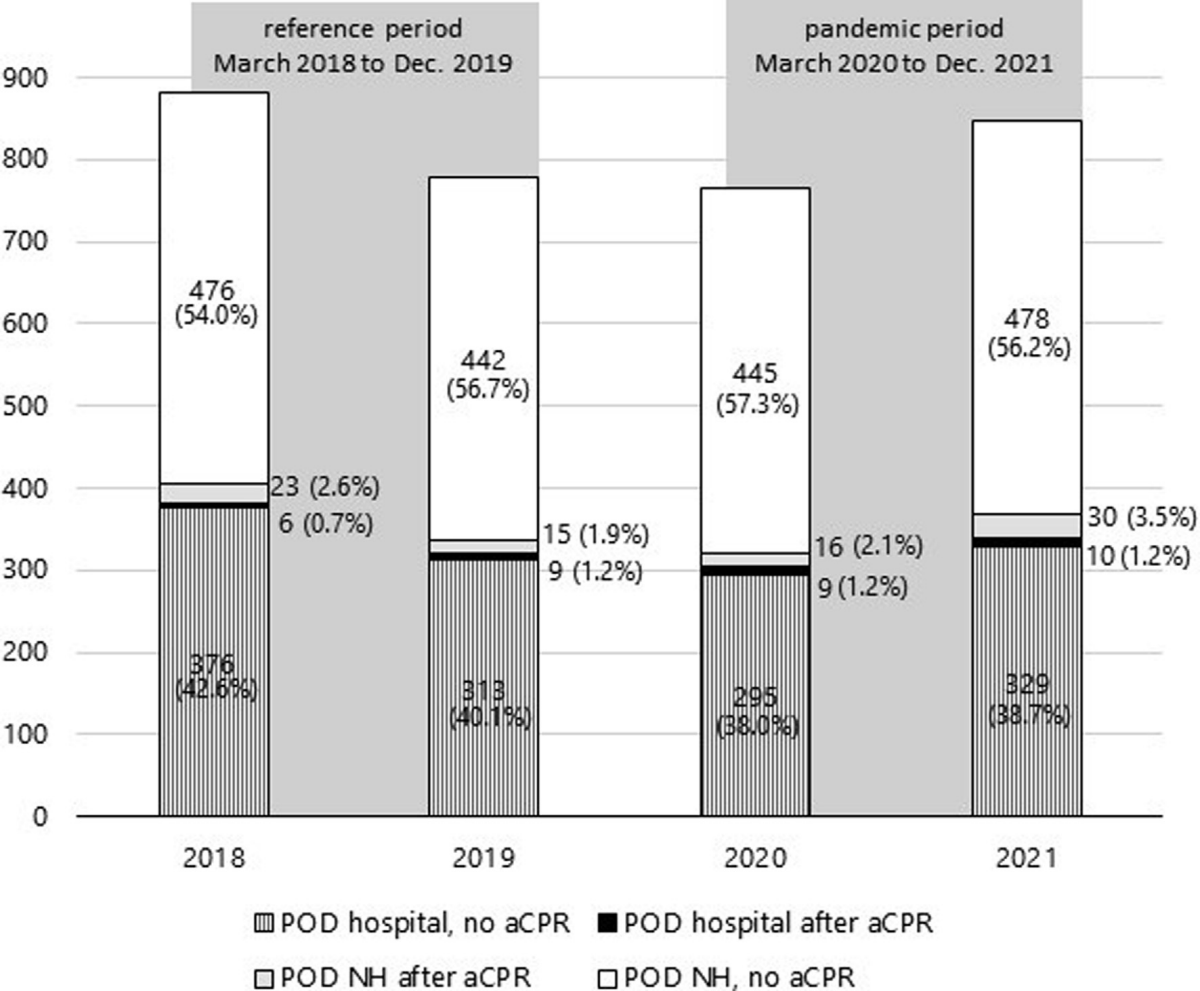
Trends in nursing home residents’ place of death and prehospital resuscitation attempts. Indicated are total numbers of the full survey; PoD - place of death; aCPR - attempted cardiopulmonary resuscitation; NH - nursing home; Divergence of summation due to PoD “other”.

Of these NH residents, 118 (3.6%) were administered aCPR with the involvement of EMS personnel. The origins of their cardiac arrests were classified as cardiac in 43 (36.4%) cases, hypoxic in 20 (16.9%) cases, and “other” or not known in 55 (46.6%) cases. The initial rhythm was shockable in 11 (9.3%) individuals. Collapse was not observed in 70 (59.3%) cases; however, it was observed in 38 (32.2%) cases by nursing staff and in 10 (8.5%) cases by EMS personnel. Chest compression was initiated by nursing staff in 60 (50.8%) cases and by EMS personnel in 58 (49.2%) cases. A return of spontaneous circulation was achieved in 35 (29.7%) cases, while 84 (71.2%) individuals died at their NH and 34 (28.8%) died after transfer to hospital. None of the NH residents who underwent aCPR survived to hospital discharge. Table 1 presents the demographic data for all patients and deceased NH residents. Fig. 1 illustrates the number of aCPR, deaths, and yearly trends.

**Table 1:** Data for all deceased nursing home residents and those for whom resuscitation was attempted.

	2018	2019	2020	2021	2018–2021
All	882(26.8%)	780(23.7%)	776(23.6%)	850(25.9%)	3,288
Female	607(68.8%)	522(66.9%)	507(65.3%)	560(65.9%)	2,196(66.8%)
Mean age in years (*SD*)	87(8.6)	86(8.7)	87(8.3)	86(8.8)	87(8.6)
Context of aCPR	29(3.3%)	24(3.1%)	25(3.2%)	40(4.7%)	118(3.5%)
Female	15(51.7%)	15(62.5%)	14(56.0%)	24(60.0%)	68(57.6%)
Mean age in years (*SD*)	86(7.3)	87(7.4)	85(8.9)	85(7.3)	86(7.6)

*SD* - standard deviation; aCPR - attempted cardiopulmonary resuscitation.

### Comparision between reference and pandemic period

Fig. 1 displays the timeframe between the reference and the pandemic periods. During the reference period (i.e., prior to the Covid-19-pandemic), 1,485 NH residents died, including 50 (3.4%) in the context of aCPR. Of these, 863 (58.1%) died at the PoD “NH” with 36 (4.2%) dying during aCPR. Additionally, 620 (41.8%) died at the PoD “hospital” including 14 (2.3%) following prehospital aCPR. The mean age was 87 years (*SD* ± 8.6), and 1,006 (67.7%) were female.

During the pandemic period, 1,475 NH residents died, including 60 (4.1%) in the context of aCPR. Of these, 891 (60.4%) died at the PoD “NH” with 43 (4.8%) dying during aCPR. Additionally, 574 (38.9%) died at the PoD “hospital” including 17 (3.0%) following prehospital aCPR. The mean age was 87 years (*SD* ± 8.5), and 969 (65.7%) were female.

The number of deaths per bed per year was 0.26 during both time periods. However, over the 4-year study period, the number of deaths per bed per year rose from 8.8 to 10.9. The ratio of resuscitation attempts to deaths also increased from 5.8% to 7.1% (RD 1.3%). Table 2 shows the demographic data and results for the reference and pandemic periods for all deceased residents and subgroups, according to sex.

**Table 2:** Data for the reference and pandemic periods for the entire study population, and female and male subgroups.

		**Reference period**	**Pandemic period**
**All deceased NH residents**		1,485	1,475
	PoD “NH”	863 (58.1%)	891 (60.4%)
	PoD “hospital”	620 (41.8%)	574 (38.9%)
	PoD “other”	2 (0.1%)	10 (0.7%)
	Context of aCPR	50 (3.4%)	60 (4.1%)
	Mean age in years (*SD*)	87 (8.6)	87 (8.5)
Female		1006 (67.7%)	969 (65.7%)
	PoD “NH”	601 (59.7%)	612 (63.2%)
	PoD “hospital”	404 (40.2%)	349 (36.0%)
	PoD “other”	1 (0.1%)	8 (0.8%)
	Context of aCPR	30 (3.0%)	36 (3.7%)
	Mean age in years (*SD*)	88 (8.0)	88 (7.7)
Male		479 (32.3%)	505 (34.2%)
	PoD “NH”	262 (54.7%)	279 (55.2%)
	PoD “hospital”	216 (45.1%)	224 (44.4%)
	PoD “other”	1 (0.2%)	2 (0.4%)
	Context of aCPR	20 (4.2%)	25 (5.0%)
	Mean age in years (*SD*)	83 (9.0)	84 (9.2)

PoD - place of death; NH - nursing home; aCPR - attempted cardiopulmonary resuscitation; *SD* - standard deviation.

### Sex differences during the reference and pandemic periods

During the reference period, 1,006 (67.7%) of the deceased NH residents were female, of whom 601 (59.7%) died in the NH and 404 (40.2%) died in hospital. Thirty (3.0%) of these female NH residents underwent aCPR. The remaining 479 (32.3%) were male, of whom 262 (54.7%) died in the NH and 216 (45.1%) died in hospital. Twenty (4.2%) of these male NH residents underwent aCPR.

Throughout the pandemic period 969 (65.7%) of the deceased NH residents were female, of whom 612 (63.2%) died in the NH and 349 (36.0%) died in hospital. Thirty-six (3.7%) of these female NH residents underwent aCPR. The remaining 505 (34.2%) were male, of whom 279 (55.2%) died in the NH and 224 (44.4%) in hospital. Twenty-five (5.0%) of these male NH residents underwent aCPR.

Among the female NH residents who died at the NH, the ratio of resuscitation attempts to deaths increased from 5.0% during the reference period to 5.9% during the pandemic period (RD 0.9%). In male residents the risk of receiving aCPR increased from 7.6% to 9.0% (RD 1.4%).

### Nursing homes

A total of 31 NHs providing residential long-term care were identified, including one facility under non-profit ownership founded at the turn of the year 2019/2020. The average number of beds per NH was 102 (*SD* ± 39; min–max 34–210). Among these, 13 (41.9%) provided up to 90 beds, 8 (25.8%) had 91 to 120 beds, and 10 (32.3%) had more than 120 beds. Nineteen (61.3%) of the facilities were under non-profit ownership, five (16.1%) were owned privately on a nationwide scale, and seven (22.6%) were privately owned on a local scale. None was in public ownership.

**Fig. 2:**
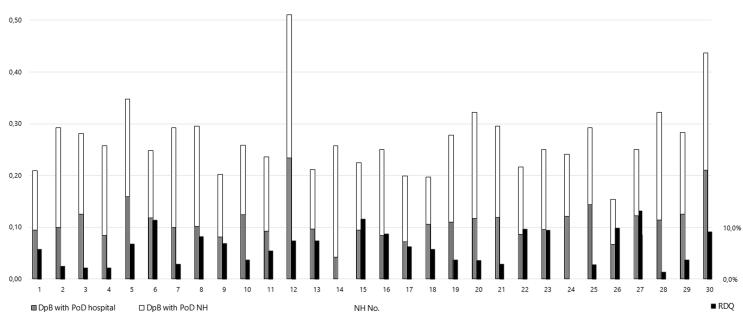
Incidence of resuscitation attempts and absolute risk of receiving futile resuscitation in different nursing homes over the 4-year period. DpB - deaths per bed per year; PoD - place of death; NH - nursing home; RDQ - resuscitation attempts without survival to hospital discharge per deaths at the PoD “NH”.

Over the 4-year study period the average number of deceased residents across the different NHs was 106 (±51; 36–292), resulting in 0.27 (±0.07; 0.15–0.51) deaths per bed per year. The average number of aCPR events was 3.8 (±3; 0–11), with 9.5 (±5.5; 0–21.1) aCPR events per 1,000 beds per year. The average ratio of aCPR to deaths was 6.0% (0–12.5%). Fig. 2 displays the differences in PoD and aCPR across the 30 NHs that were operational throughout the 4-year study period.

During the reference period the average number of deaths per bed per year in the different NHs ranged from 0.14 to 0.49, with an average of 0.26 (SD ± 0.07). The percentage of PoD “NH” averaged 59.9% (37.7– 85.7%). Finally, there was an average of 8.1 (±6.5; 0–19.7) aCPR attempts per 1,000 beds per year, and an average ratio of aCPR events to deaths of 5.8% (0–15.0%).

Throughout the pandemic period, the average number of deaths per bed per year was 0.26 (±0.07; 0.14–0.53). The percentage of PoD “NH” was 60.3% (44.8–77.8%). A total of 10.9 (±8.3; 0–34.1) aCPR events per 1,000 beds per year occurred, and the average ratio of resuscitation attempts to deaths was 7.1% (0–25.0%). Table 3 shows the PoD and aCPR data for the 30 NHs during the reference and pandemic periods.

**Table 3:** Data for the different nursing homes during the reference and pandemic periods.

			**Reference period**				**Pandemic period**				
NH	ownership	No. beds	DpB	PoD NH	RpB	RDQ	DpB	PoD NH	RpB	RDQ	RD for futile aCPR
1	private-nationwide	91 to 120	0.29	58.6%	14.7	8.8%	0.14	44.8%	0	0	−8.8%
2	non-profit	91 to 120	0.32	58.6%	5.5	2.9%	0.24	65.9%	5.5	3.4%	0.5%
3	non-profit	up to 90	0.27	62.9%	0	0	0.26	50.0%	7.6	5.9%	5.9%
4	private-local	up to 90	0.23	71.4%	8.4	5.0%	0.24	69.0%	0	0	−5.0%
5	non-profit	over 120	0.39	54.0%	15.6	7.4%	0.28	52.3%	13.0	8.8%	1.4%
6	non-profit	over 120	0.27	57.3%	14.5	9.3%	0.21	49.2%	14.5	13.8%	4.5%
7	non-profit	over 120	0.30	56.3%	4.2	2.5%	0.28	77.3%	4.2	2.0%	−0.5%
8	non-profit	over 120	0.25	62.0%	9.7	6.1%	0.34	68.6%	25.8	11,1%	5.0%
9	private-local	up to 90	0.22	58.3%	6.1	4.8%	0.20	63.6%	6.1	4.8%	0.0%
10	private-local	over 120	0.26	37.7%	7.5	7.7%	0.24	69.2%	3.8	2.2%	−5.5%
11	private-nationwide	over 120	0.19	61.8%	3.5	2.9%	0.28	60.0%	14.0	8.3%	5.4%
12	private-local	up to 90	0.38	69.7%	11.4	4.3%	0.53	51.1%	34.1	12.5%	8.2%
13	private-nationwide	91 to 120	0.19	50.0%	14.1	15.0%	0.25	60.4%	4.7	3.1%	−11.9%
14	non-profit	up to 90	0.22	85.7%	0	0	0.29	77.8%	0	0	0.0%
15	non-profit	over 120	0.22	64.2%	16.8	11.8%	0.21	51.0%	12.6	12.0%	0.2%
16	non-profit	up to 90	0.24	73.3%	16.0	9.1%	0.27	67.6%	16.0	8.7%	−0.4%
17	private-nationwide	over 120	0.21	66.0%	13.4	9.7%	0.18	67.5%	4.5	3.7%	−6.0%
18	private-nationwide	up to 90	0.16	50.0%	0	0	0.22	52.6%	11.6	10.0%	10.0%
19	non-profit	91 to 120	0.24	65.4%	0	0	0.28	56.7%	14.1	8.8%	8.8%
20	private-local	up to 90	0.33	67.5%	8.3	3.7%	0.32	61.5%	8.3	4.2%	0.5%
21	non-profit	91 to 120	0.31	54.7%	0.0	0.0%	0.29	64.0%	11.6	6.3%	6.3%
22	private-local	over 120	0.24	62.7%	9.2	6.3%	0.21	52.2%	23.1	20.8%	14.6%
23	non-profit	up to 90	0.28	65.1%	19.5	10.7%	0.24	56.8%	13.0	9.5%	−1.2%
24	non-profit	up to 90	0.23	48.0%	0	0	0.25	53.6%	0	0	0.0%
25	private-local	up to 90	0.28	50.0%	0	0	0.29	61.3%	9.2	5.3%	5.3%
26	non-profit	91 to 120	0.14	60.0%	0.0	0.0%	0.15	62.5%	23.7	25.0%	25.0%
27	non-profit	over 120	0.25	48.4%	12.1	10.0%	0.24	52.5%	12.1	9.7%	−0.3%
28	non-profit	up to 90	0.31	61.7%	6.6	3.4%	0.33	70.0%	0	0	−3.4%
29	non-profit	up to 90	0.21	68.8%	6.7	4.5%	0.25	56.8%	6.7	4.8%	0.2%
30	non-profit	91 to 120	0.49	45.3%	19.7	8.8%	0.37	64.3%	19.7	8.3%	−0.5%
Cohort of all nursing home residents			0.26	58.1%	8.8	5.8%	0.26	60.4%	10.9	7.1%	1.3%

DpB - deaths per bed per year; PoD - place of death; NH - nursing home; RpB - Resuscitation attempts per 1,000 beds per year; RDQ - resuscitation attempts without survival to hospital discharge per deaths at PoD “NH”; RD - risk difference; aCPR – attempted cardiopulmonary resuscitation.

## Discussion

The present research surveyed the aCPR events and PoDs of residents of the different NHs in an urban district in Germany. The results provide a picture of aCPR overuse and underuse in these facilities and a broad view on the end of life situation of NH residents. Furthermore, the research compared periods just before and during the Covid-19 pandemic.

### The significance of different indicators

Data from diverse populations can be used to construct indicators. However, the application of such indicators as a direct measure of quality or for benchmarking purposes is questionable. Quality assessment in long-term care necessitates a systemic understanding of quality.^22^ In this context, the results do not provide a direct assessment of quality of care, but rather serve as a starting point for more focused investigations. Of note, PoD is the most commonly used metric for assessing end-of-life quality, but it provides no indication of the quality of care or patient’s experience in the specific setting.[14], [15], [23]

To address the potential underuse of aCPR, the present analysis standardized deaths and aCPR events based on the number of beds in each NH (i.e., NH population size). Additionally, the ratio of aCPR events to deaths provided insight into potential overuse,[17], [20] representing the absolute risk of receiving aCPR at end of life. Of note, epidemiological resuscitation research has not primarily focused on overuse,^24^ making the absolute risk of futile aCPR a valuable contribution to the literature.

### The cohort of all deceased NH-residents

Regarding the entire cohort of deceased NH residents, the number of deaths varied annually but did not increase during the pandemic period. However, the incidence of aCPR increased from 8.8 during the reference period to 10.9 during the pandemic period. The absolute risk of receiving futile aCPR for residents who died at the NH increased by 1.3%. The demographic data showed little to no variation. Considering the study region as a whole, the results do not indicate that NH residents were significantly discouraged from being transferred to hospital. While the balance between aCPR overuse and underuse did shift slightly towards overuse during the pandemic, it is essential to note that, during the pandemic, excess mortality was not observed in Braunschweig, in contrast to the general population of Lower Saxony and Germany more widely.[20], [25] This suggests that the study region experienced fewer direct and indirect effects of the pandemic, potentially limiting the extent to which the results may apply to other regions—especially those that were strongly affected by the pandemic.

The majority of the deceased NH residents were female. Compared to their male counterparts, these female residents died at an older age, more frequently at the NH and less frequently in the context of aCPR. During the pandemic, the sex related difference in the balance between aCPR overuse and underuse at the end of life increased: RD for futile aCPR was 0.9% for female residents and 1.4% for male residents. Sex-related differences in the situations, treatments and outcomes of cardiac arrest have previously been reported.[26], [27] The present results indicate that such sex-related differences may also manifest in a controlled setting with continuous nursing care.

However, the underlying reasons for these differences remain unclear. The data sample did not provide insight into individual cases, including whether transportation to the hospital (or aCPR) was not indicated, declined by the patient, or simply omitted by health care providers.

### Variations among nursing homes

Significant variations were observed between different NHs. During the 4-year study period, the incidence of death varied by a factor of more than three, and aCPR incidence ranged from 0 to more than 20 per 1,000 beds per year. Moreover, the absolute risk of receiving aCPR at end of life ranged from 0% to 12.5% across the different facilities. Fig. 2 illustrates the substantial variations in the number of deaths per bed per year, residents’ PoD, and residents’ absolute risk to undergo aCPR at end of life across the 30 NHs studied over the 4-year period.

In the comparision of the reference and pandemic period, specific facilities demonstrated significant changes. For instance, facility no. 1 experienced half as many incidents of death and a decrease in the number of residents passing away at the NH. Additionally, in this facility, no aCPR was conducted during the pandemic, while 15 aCPR per 1,000 beds per year were conducted prior to the pandemic. Moreover, the absolute risk of futile aCPR decreased (-9%). In contrast, facility No. 26 showed no substantial changes in the place and incidence of death, but the incidence of aCPR and the absolute risk of futile aCPR increased (RD 25%). Table 3 presents the relevant data for all 30 NHs. The wide-ranging variations in aCPR incidence between NHs could be considered a significant factor for multi-level modeling in future research.

There are numerous potential causes of the substantial differences and contrasting trends that were observed across facilities, including the differing medical indications and individual care preferences of NH residents, as well as differences in NH policies, staff competence and availability, access to physicians and other factors.[6], [22], [28], [29].

### Strengths and limitations

The present study is unique in its analysis of aCPR in relation to deaths in NH residents over a 4-year period, shedding light on the potential overuse and underuse of aCPR. One strength of the research is the complete data collection for the full survey. However, it is important to also acknowledge the limitations of this research, which stem from the retrospective analysis of data. Furthermore, information about the different NHs was limited, and data regarding individual cases were not available. Finally, the main limitation refers to the questionable generalizability of the results from a single urban district in Germany to broader populations.

## Conclusions

The frequency with which aCPR is administered to dying NH residents is generally low. However, the present study revealed significant variation between NHs with regard to PoD and the overuse and underuse of aCPR. EMS personnel and researchers should be aware that, in NHs, residents receiving aCPR represent only a small minority of the residents who pass away in these settings.

## Declaration of interest and funding

The “NOVELLE” project received funding from the Innovation Fund of the German Federal Joint Committee (G-BA), under Grant No. 01NVF18007. The funding body was not involved in the execution of the present study. With the exception of the affiliations and funding mentioned, the authors declare no further conflicts of interest.

## Declaration of competing interest

The authors declare that they have no known competing financial interests or personal relationships that could have appeared to influence the work reported in this paper.
